# The study on the effects of gamified interactive e-books on students’ learning achievements and motivation in a Chinese character learning flipped classroom

**DOI:** 10.3389/fpsyg.2023.1236297

**Published:** 2023-08-04

**Authors:** Chuang Chen, Nurullizam Jamiat, Yongchun Mao

**Affiliations:** ^1^School of Art and Design, Zhengzhou University of Industrial Technology, Zhengzhou, China; ^2^Centre for Instructional Technology and Multimedia, Universiti Sains Malaysia, Penang, Malaysia; ^3^School of Arts and Design, Qilu University of Technology (Shandong Academy of Sciences), Jinan, China

**Keywords:** flipped classroom, Chinese character learning, gamified interactive e-books, motivation, learning achievement, gamification in education, educational technologies

## Abstract

Existing empirical research has demonstrated the positive effects of flipping the classroom to improve student motivation and achievement by flipping in-class learning content to pre-class. However, the flipped classroom approach requires that students be engaged and motivated in the pre-class stage to ensure that the in-class learning activities run smoothly. Previous studies have highlighted the difficulties that students often encounter when trying to learn Chinese characters in Chinese language classes, especially those who are in the early stages of learning the language. Therefore, in this study, a gamified interactive e-book was developed and used in a flipped classroom for Chinese character learning. To evaluate the effectiveness of this approach, a pre-test and post-test control group experimental design was used. The participants were 90 s-grade students from a public primary school in Zhengzhou, China, who were randomly assigned to two experimental groups and one control group: the students who used a gamified interactive e-book in a Chinese character learning flipped classroom (GIEFC group), the students who used a traditional flipped classroom (TFC group), and the students who used a traditional teaching classroom (TTC group). The experimental results indicated that students in the GIEFC group scored higher than those in the TFC and TTC groups in terms of learning achievements and motivation. In addition, the experimental results also demonstrated the positive effects of gamified interactive e-books in flipped classroom learning. Future research could explore a variety of different types of game elements as well as the extension of research to other subjects.

## 1. Introduction

The field of language studies aims to provide students with a deep understanding of the structure and use of a particular language as well as an appreciation of the literature of a particular time and place (Katz, 1964). Chinese language courses are designed to provide students with the ability to understand and master the structure, sounds, and forms of the language while also fostering an understanding of language and literature in the context of specific social and cultural environments (Cotterall, 2000). These language courses are comprehensive and practical, emphasizing the development of listening, speaking, reading, and writing skills as well as the critical interpretation of literary works and forms within specific cultural contexts (Kubler, 2018). Several scholars have highlighted the difficulties that students often encounter when attempting to acquire Chinese character knowledge in Chinese language classes, especially those who are in the early stages of learning the language because Chinese characters are the basic symbols that make up the Chinese language and belong to the syllabic group of ideographic characters (Qian and McCormick, 2014; Kubler, 2018). Traditional Chinese language courses adopt an instructional approach that emphasizes the one-way transfer of knowledge from teachers to students, which results in limited opportunities for students to actively apply or practice their acquired knowledge (Thoms et al., 2017). There is a possibility that students’ learning performance may be adversely affected (Wen, 1997).

The flipped classroom instructional approach is designed to prioritize student-centered learning by shifting the delivery of learning content from in-class to pre-class, thus creating more opportunities for students to practice skills such as problem solving, laboratory work, and discussion during class (Akçayır and Akçayır, 2018). The flipped classroom approach is a technological and pedagogical support for learning strategies that consists of two phases: pre-class, where students can access learning content through technology, and in-class, where interactive group learning activities are performed in the classroom (Bishop and Verleger, 2013). Therefore, it is important for students to understand and focus on the material prior to class. Meanwhile, the flipped classroom approach allows students to reinforce their understanding of the material, identify gaps in their understanding, and take the necessary steps to ensure sustained knowledge acquisition (Hung, 2015; Chuang et al., 2018). Some researchers have recognized that traditional classroom methods are lacking in practice and have begun to address this issue by using flipped classroom methods (Evseeva and Solozhenko, 2015; Hung, 2017).

As students prepare for class using the various methods provided by the teacher, either on the computer or tablet, they can learn at their own pace, such as pause, start, repeat, etc. (Chen Hsieh et al., 2017). Therefore, in order to achieve the desired learning outcomes in the flipped classroom, students must have sufficient prior knowledge, adequate study skills, and a high level of self-discipline (Chang et al., 2022). Some studies indicate that due to the complexity of language learning, the motivation of many students suffers, leading to poor learning performance (Aşıksoy, 2018). Since the flipped classroom approach requires students to study before class, this additional learning process and the complexity of language learning itself may lead to resistance from some students (Zainuddin, 2018). In addition, due to a lack of self-discipline and motivation, students may not be able to dedicate a reasonable amount of time to watching the videos and understanding the learning content during the pre-class learning activities. In such cases, they are likely to be unable to learn effectively from the in-class activities (Lai and Hwang, 2016). Furthermore, it has been noted that the use of flipped learning strategies may only be effective for highly motivated students due to the extra workload involved (Yilmaz, 2017). Therefore, to address the aforementioned challenges, it is necessary to provide students with effective learning tools or strategies prior to the beginning of classroom sessions in order to support their knowledge acquisition and the achievement of broader educational goals (Cabı, 2018; Galindo-Dominguez, 2021; Huang et al., 2022).

With the advancement of digital and mobile technologies, e-books have become increasingly popular for educational purposes (Bus et al., 2015). E-books are self-contained digital texts with a structure similar to traditional paper books that can be read on an electronic device (Kong et al., 2018). The main difference between e-books and paper books is the technology-based features available in e-books, such as multimedia and interactive features (Korat and Falk, 2019; Chuang and Jamiat, 2023). These e-books with interactive features are typically referred to as interactive e-books. The use of such e-books in the language classroom can have significant benefits as they offer immersive and interactive approaches that embed learning content in realistic contexts (Takacs et al., 2015; Chen and Jamiat, 2023).

The use of gamification as a learning strategy has attracted significant attention and interest in both practice and academic research. It is characterized as “the use of game design elements in non-game contexts” (Deterding et al., 2011), with education being one of the main areas of gamification research. By incorporating game elements into learning content through gamified learning strategies, students can be provided with a realistic simulation or learning environment that allows them to have an enjoyable learning experience (Subhash and Cudney, 2018), thus stimulating their interest in learning and enhancing their motivation, learning perception, and effective learning reflection (Hassan et al., 2021). Moreover, gamified learning is an organic integration of gamification and classroom teaching, and the use of gamification in learning and teaching is considered to be highly engaging for learners, stimulating motivation, learner engagement, and social impact (Zainuddin et al., 2020).

In this study, gamified interactive e-books based on the SDT framework were developed for Chinese language learning and integrated into the flipped classroom model. The purpose of this study was to integrate a Chinese language course with gamified interactive e-books to facilitate better knowledge retention and learning, thereby improving students’ achievement and motivation to learn, which are essential in Chinese language education. The effectiveness of this approach was then evaluated through an experiment conducted in a second-grade Chinese language classroom in an primary school. The research questions were formulated as follows:

1. Do students who use gamified interactive e-books in a Chinese character learning flipped classroom have better learning achievements than students who use a traditional flipped classroom and a traditional teaching classroom?
2. Do students who use gamified interactive e-books in a Chinese character learning flipped classroom have higher learning motivation than students who use a traditional flipped classroom and a traditional teaching classroom?

## 2. Literature review

### 2.1. Flipped classroom approach

According to some researchers, traditional teacher-centered teaching techniques rarely emphasize critical thinking or practical problem solving. They also claim that traditional classrooms focus on the direct delivery of content to students by the teacher (Paris and Gespass, 2001; Schuh, 2004). However, the flipped classroom, a student-centered approach, is changing traditional classroom teaching approaches (Chuang et al., 2018). In the flipped classroom, the role of the teacher shifts from simply delivering knowledge to effectively facilitating student learning (Limniou et al., 2018). This instructional approach not only promotes deep understanding of concepts rather than rote memorization (DeRuisseau, 2016; Burke and Fedorek, 2017), but also fosters students’ higher-order thinking skills (Hung, 2018; Liu and Zhang, 2022).

The main purpose of implementing a flipped classroom approach is to allow students to adequately prepare for course content at their individual pace and using their preferred learning style, thus creating more opportunities while improving the overall quality of face-to-face instruction and learning activities (Burke and Fedorek, 2017; Wang et al., 2018). As indicated by Yang and Chen (2020), one of the benefits associated with flipped classrooms is increased student motivation. Typically, students view the flipped classroom as a more attractive option than traditional classrooms, so they are more likely to diligently follow the instructional videos provided by their instructors (Heijstra and Sigurðardóttir, 2018). This allows them to identify the problems they do not understand at the pre-class stage and seek guidance from teachers or discuss them with peers at the in-class stage (Lee and Choi, 2019).

Another benefit of the flipped classroom is that students have the autonomy to learn, allowing them the freedom to learn by accessing video courses at their own convenience (Yoon et al., 2020). This approach allows teachers to focus more instructional time on developing meaningful activities such as group discussions, practices, and problem solving while providing students with more opportunities to develop their independent learning and problem solving skills (Al-Zahrani, 2015). Ultimately, this approach promotes a more comprehensive understanding of subject matter content and makes students more successful in their learning (Lee et al., 2022).

Given the advantages of the flipped classroom, some research has begun to apply this approach to a variety of disciplines and educational levels (Strelan et al., 2020). For the language classroom, researchers have conducted various studies, but have obtained inconsistent results. For example, Turan and Akdag-Cimen’s (2020) systematic literature review confirms that flipping in EFL classrooms helps learners increase their engagement and improve their speaking skills, peer interaction, and overall learning. Consistent with Turan and Akdag-Cimen’s (2020) research, Zou et al. (2022) found that flipped language classrooms not only resulted in improved learning achievement and increased motivation among learners, but also contributed to the development of their self-regulation skills, self-confidence, and higher-order cognition abilities. Yang et al. (2018) conducted a study to compare the effectiveness of the traditional classroom method and the flipped classroom method for teaching Chinese as a foreign language. They collected data from two first-year Chinese language courses, and found that students in the flipped classroom had significantly better performance in speaking skills and learning outcomes. In contrast, some researchers have reported no effect of the flipped classroom on students’ academic achievement and motivation. For example, Butzler’s (2014) dissertation compared traditional and flipped classrooms and found no differences in students’ motivation, learning achievement, or satisfaction. Consistent with Butzler (2014) and Cabı’s (2018) study focused on investigating the impact of the flipped classroom model on students’ academic achievement, and after four weeks of the experiment, the results of the study showed that there were no statistically significant differences between the groups.

What is more, based on a critical review of the challenges of flipped classrooms in language education, Lo and Hew (2017) claimed that the use of flipped classroom methods in language education had a neutral or small impact on student achievement compared to traditional classrooms. In contrast, students’ attitudes and motivation to learn in flipped classrooms had different results due to the extra workload and technology issues (Lo and Hew, 2017; Turan and Akdag-Cimen, 2020). According to previous research, it has been observed that students may face challenges in sustaining their attention to videos provided by instructors as part of pre-class learning (Lee and Choi, 2019; Deng and Gao, 2023) and resist learning the topics on their own outside the classroom in the FC Model (Cabı, 2018; Nja et al., 2022). Lee and Choi (2019) state that students can be biased and resistant to this unfamiliar approach. Meanwhile, Poorly trained instructors and poor video quality have reduced the effectiveness of the approach (Zou et al., 2022). As a result, some researchers argue that without appropriate instruction and strategies, students in conditioned learning environments may exhibit poor achievement behaviors and higher levels of learning anxiety (Howitt and Pegrum, 2015; Guo, 2019; Doğan et al., 2023).

Surprisingly, our literature search revealed few studies that incorporated mobile technology into the flipped classroom. Thus, this study tried to search again using a combination of the keywords technology and flipped classroom, and although more results appeared, we still found some problems: (1) Studies on the flipped classroom mostly focused on English language learning, with few on Chinese character learning courses; (2) the flipped classroom was mostly applied to second or foreign language teaching; (3) the participants were mostly university students, with few pre-university participants, especially primary school students; (4) most studies focused on engineering, mathematics, computer programming, physics, and medicine; (5) most studies were conducted in the United States, Canada, and Taiwan, with fewer in China. Meanwhile, Hwang et al. (2009) highlighted the effectiveness of using mobile technology to bridge learning activities outside and inside the classroom. Therefore, the main focus of this study was to develop an appropriate mobile technology approach to help students learn effectively in a flipped Chinese classroom.

### 2.2. Gamification, motivation, and flipped language classroom

The implementation of gamification and game-based learning techniques through mobile technology has gained popularity as a means to improve learning outcomes (Lavoué et al., 2018). Gamification refers to the use of game elements in a non-game context, while game-based learning involves the creation of an entire game to promote specific educational objectives (Deterding et al., 2011). The gamification process involves modifying existing learning strategies by incorporating game elements such as achievements, avatars, badges, collections, gifting, leader boards, points, virtual goods, and levels to stimulate engagement, problem solving, and the achievement of desired learning outcomes (Werbach et al., 2012; Zainuddin et al., 2020). In summary, the concept of gamification involves removing the underlying foundations of a game while retaining its core structural elements, including rules, output, feedback, interaction, challenge, storyline, and goals (Seaborn and Fels, 2015). In their study, Werbach et al. (2012) classified the game design elements that create gamified scenes into three categories: dynamics, mechanics, and components, as shown in Table 1.

**Table 1:** Three categories of game design element.

Categories	Definition	Contents
Dynamics	Represents the highest conceptual level in a gamified system	Constraints, emotions, narrative, progression and relationships.
Mechanics	Refer to the elements that move the action forward	Challenges, chance, competition, cooperation, feedback, resource acquisition, rewards.
Components	The basic level of the gamification process and encompass the specific instances of mechanics and dynamics	Achievements, avatars, badges, collections, content unlocking, gifting, leaderboards, levels, points, virtual goods, etc.

Empirical studies have shown that gamification is gaining recognition as an effective learning strategy, and recent studies have demonstrated the potential of gamification to increase student engagement, motivation, academic performance, and social influence (Subhash and Cudney, 2018; Zainuddin, 2018; Zhao et al., 2023). In addition, students can have immersive learning experiences when exposed to gamification. According to Ekici’s (2021) systematic literature review of gamification in flipped learning, research shows that incorporating gaming elements into a flipped classroom increases motivation, participation, and educational outcomes. Ho’s (2020) study confirmed previous findings that students’ behavioral, cognitive, and motivational engagement could be enhanced through a gamified flipped classroom. Through a pilot study designed to examine students’ learning performance and perceived motivation between a gamified flipped classroom and a non-gamified flipped classroom instructional model, Zainuddin (2018) found that students’ post-test scores were higher in the gamified flipped classroom setting than in the non-gamified flipped classroom. Eryiğit et al. (2021) studied international students in an introductory Turkish language course who used a gamified mobile application for 3 weeks, which resulted in positive perceptions and effectiveness for their learning process.

Although many empirical studies have demonstrated the effectiveness of gamified instruction in education, several limitations have been identified. For example, a systematic review conducted by Zainuddin et al. (2020) in Educational Research Review found that the failures of gamified instruction are closely related to the use of game elements, instructional design, and technological issues (Zainuddin et al., 2020). Simply using game elements such as leaderboards, badges, and scores to stimulate external motivation may not guarantee student motivation and achievement (Kyewski and Krämer, 2018). Despite the debate about external rewards to stimulate intrinsic motivation, researchers have suggested that the success of gamified classrooms, both traditional and flipped, depends largely on teachers’ ability to effectively select game elements that promote meaningful learning and align with specific learning goals and instructional content (Putz et al., 2020). In addition, some researchers have raised concerns about the gaming applications used in gamified flipped classrooms, arguing that the over-inclusion of gamified elements may be detrimental to student learning, creating a trend toward e-learning and even game addiction (Young et al., 2012; Zainuddin et al., 2020). Meanwhile, Hung (2018) notes that not all course content can be transformed into a gamified flipped classroom; students still need to acquire knowledge through conventional teaching methods most of the time.

The literature review highlights the benefits and challenges associated with gamification and the flipped classroom. To address issues such as low motivation or resistance to new learning methods during the pre-course phase, researchers have incorporated mobile technologies into the flipped classroom. For example, Wang (2016) suggested that integrating ubiquitous mobile learning technologies with flipped classroom strategies could lead to self-regulated learning. Louhab et al. (2018) developed an Android mobile application for the flipped classroom and showed that using contextual dimensions, especially device context, in adaptive mobile learning is more beneficial for learners, especially in the flipped classroom. Wang and Jou (2020) emphasized the importance of using mobile learning technologies to integrate pre-class learning activities with in-class learning activities in the flipped classroom.

Therefore, this study has developed a gamified interactive e-book (hereafter, the GIEB) for the Chinese character learning flipped classroom with the purpose of improving learning achievements and motivation. The Chinese character learning module of the Chinese curriculum was selected as the design content. It is expected that a well-designed GIEB can serve as a valuable learning strategy to enhance academic performance and motivation.

## 3. Designing a GIEB for learning Chinese characters

### 3.1. Systerm structure of the GIEB

**Figure 1:**
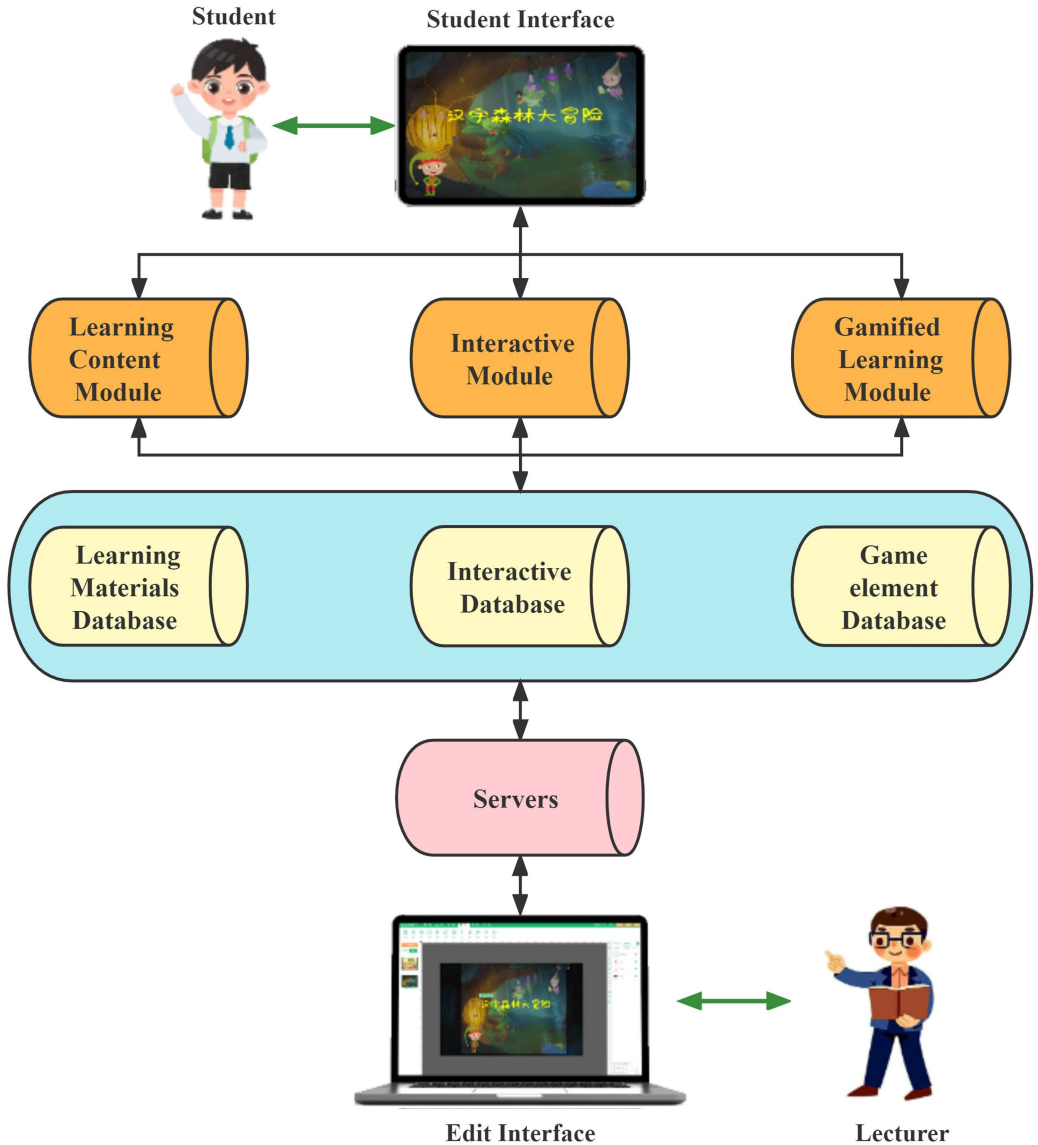
Systerm structure of the GIEB.

In this study, Yoya, an interactive course development software developed by Xiamen Elegant Web Technology Co., was used to develop CIEB for the Chinese character learning module, which is important for the students. The CIEB consists of three modules: the Learning Content Module, the Interactive Module, and the Gamified Learning Module. Each module is supported by its own database. All data is stored on Yoya Software’s servers. As shown in Figure 1.

The primary function of the learning content module is to provide materials for Chinese character learning. This module provides a virtual story as a context that enhances knowledge acquisition. The interactive module consists of a series of interactive activities that students perform using IPad, including relevant interactive actions (e.g., tasks, games) based on learning content. In the gamified learning module, game elements such as badges, scores, virtual gifts, and leaderboards are used to support learning. Although the three modules are introduced individually, each module shares content and reinforces the others.

### 3.2. SDT-based GIEB design framework

Several scholars have attempted to illustrate the connection between gamification and learning by introducing theories and frameworks (Yamani, 2021). In particular, self-determination theory (SDT) has proven effective in both gaming and gamification contexts as a means of revealing this connection (Ryan and Deci, 2020). Self-determination theory (SDT) is a motivational theory that emphasizes the importance of basic psychological needs such as competence, autonomy, and relatedness (Vallerand, 2000). Satisfying these needs is central to intrinsic motivation and, consequently, to high-quality learning. Therefore, gamifying learning can significantly improve student motivation if it successfully addresses these basic needs (Sailer and Homner, 2020). In addition, self-determination theory (SDT) emphasizes the importance of the environment in meeting these psychological needs (Ryan and Deci, 2002). Incorporating game elements into educational settings can potentially modify these environments and subsequently influence learning outcomes (Qiao et al., 2022).

**Figure 2:**
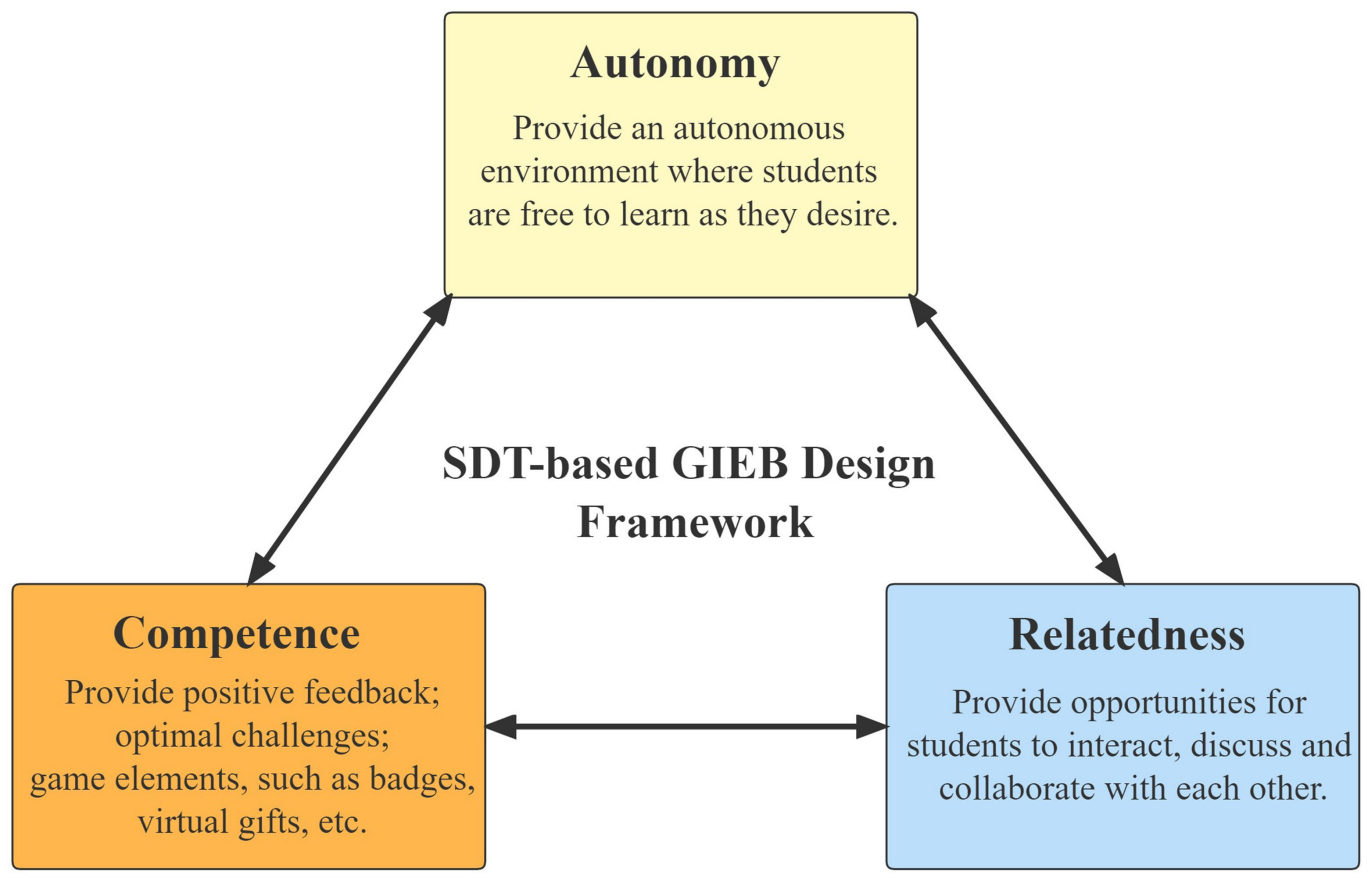
SDT-based GIEB design framework.

Lo and Hew’s (2020) research proposes that incorporating self-determination theory (SDT) into the development of gamified instructional activities is a successful approach that promotes the integration of this theoretical framework into the design of a flipped classroom. It is recommended that educators implement SDT in their instructional strategies to promote active learning and student motivation (Zepke and Leach, 2010). The psychological needs proposed by SDT to enhance student motivation, which include competence, autonomy, and relatedness (Adams et al., 2017), served as the basis for the design of the gamified interactive e-book. Thus, satisfying the three psychological needs is a key idea in SDT for maintaining intrinsic motivation and regulating extrinsic motivation (Deci and Ryan, 2008). Figure 2 illustrates how the gamified interactive e-book addresses these three psychological needs of students.

The need for competence is a reflection of the desire of human beings to effectively master their environment and to feel a sense of competence in it. For example, when students use GIEB to complete learning activities or successfully complete some tests, they can feel a sense of competence; after completing some tasks, getting rewards from the system such as badges, scores, virtual gifts, etc., or outperforming other students can also give them this feeling. The need for autonomy is satisfied when individuals have the freedom to choose and act on their own volition, identifying themselves as the source of their actions (Deci and Ryan, 2012). When learning with GIEB, students can follow their own learning patterns and styles by starting, pausing, replaying, skipping, repeating, and determining when and where to participate in learning. Relatedness is a sense of satisfaction gained from a sense of connectedness with others; it is caring for and being cared for by others, which in this study refers to student–student and student-teacher interactions (Deci and Ryan, 2008). For example, students can get peer and teacher attention and engage in learning discussions during the learning.

### 3.3. Design process of GIEB

**Figure 3:**
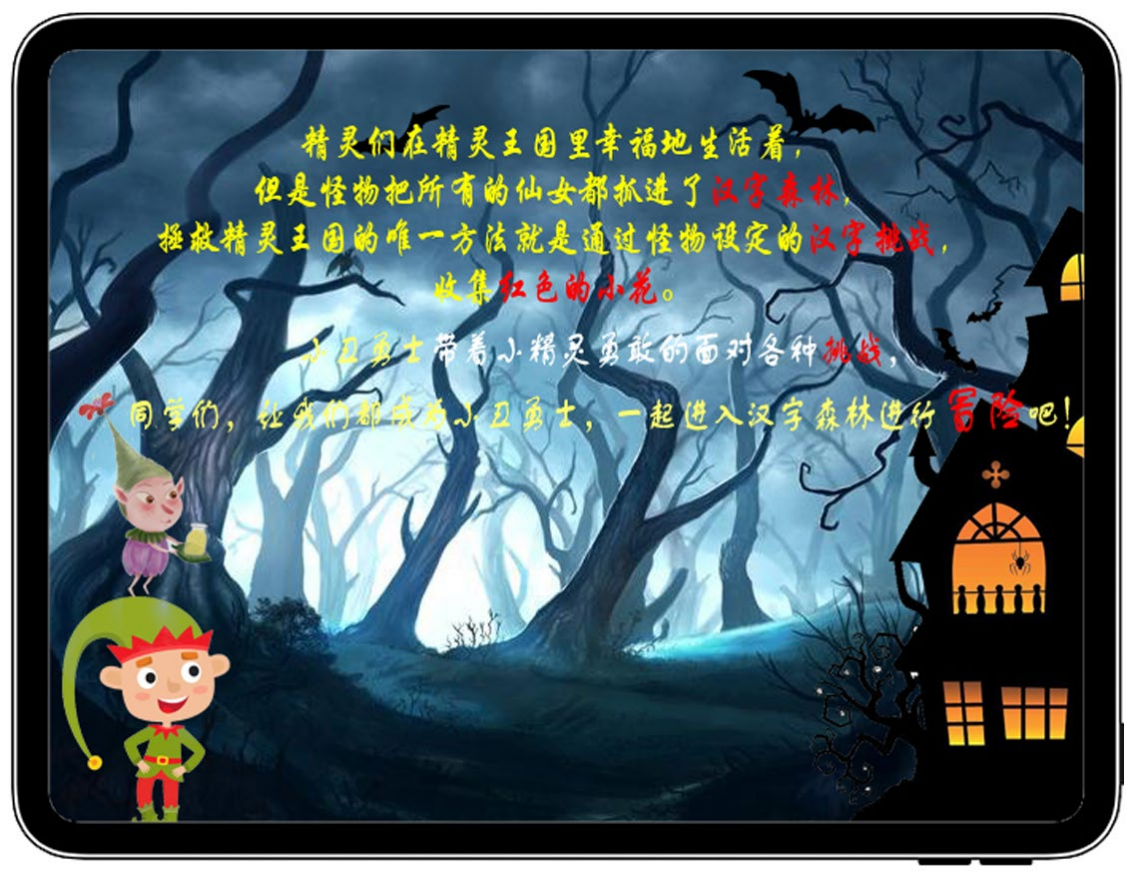
The background of story.

The GIEB was developed with a storyline that focuses on the student’s goal of acquiring knowledge about Chinese characters. The background of this story is shown in Figure 3 and serves as an initiating factor for the entire learning process, aiming to stimulate students’ curiosity and interest in the learning material. Therefore, the background of this GIEB story is that the fairies are living happily in the elves kingdom, but the monster has captured all the fairies into the Chinese character forest, and the only way to save the fairy kingdom is to pass the Chinese character challenges set by the monster and collect the red flowers. So the student-controlled avatars, Clown Warrior and Elf, embarked on a challenging journey. This e-book features six Chinese characters with real-life-related activities and games. The three learning stages include identification, learning, and writing, and students may switch between them as needed. Gamified storytelling and virtual characters allow for immersive learning experiences, promoting active and independent learning at the student’s own pace and style. This learning approach satisfies students’ need for autonomy, as their learning behaviors arise from their own volition.

**Figure 4:**
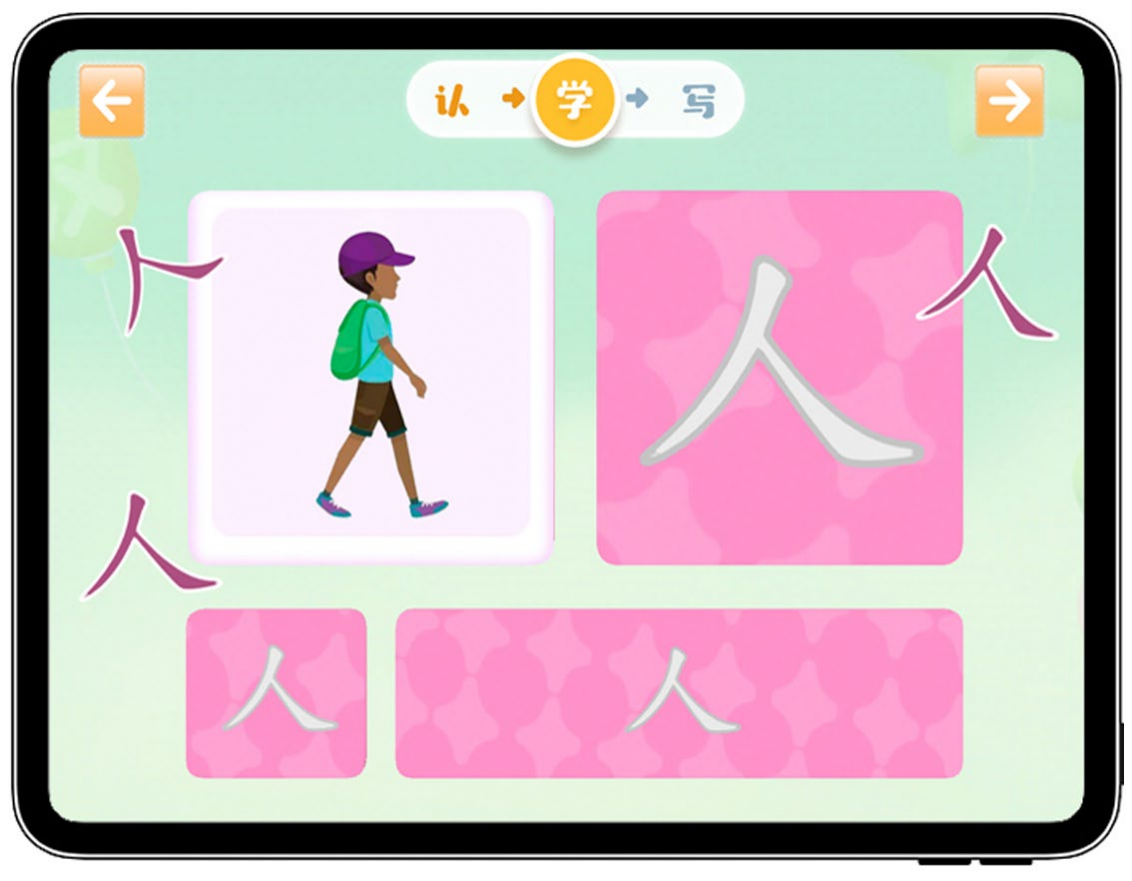
Learning content provided by GIEB.

**Figure 5:**
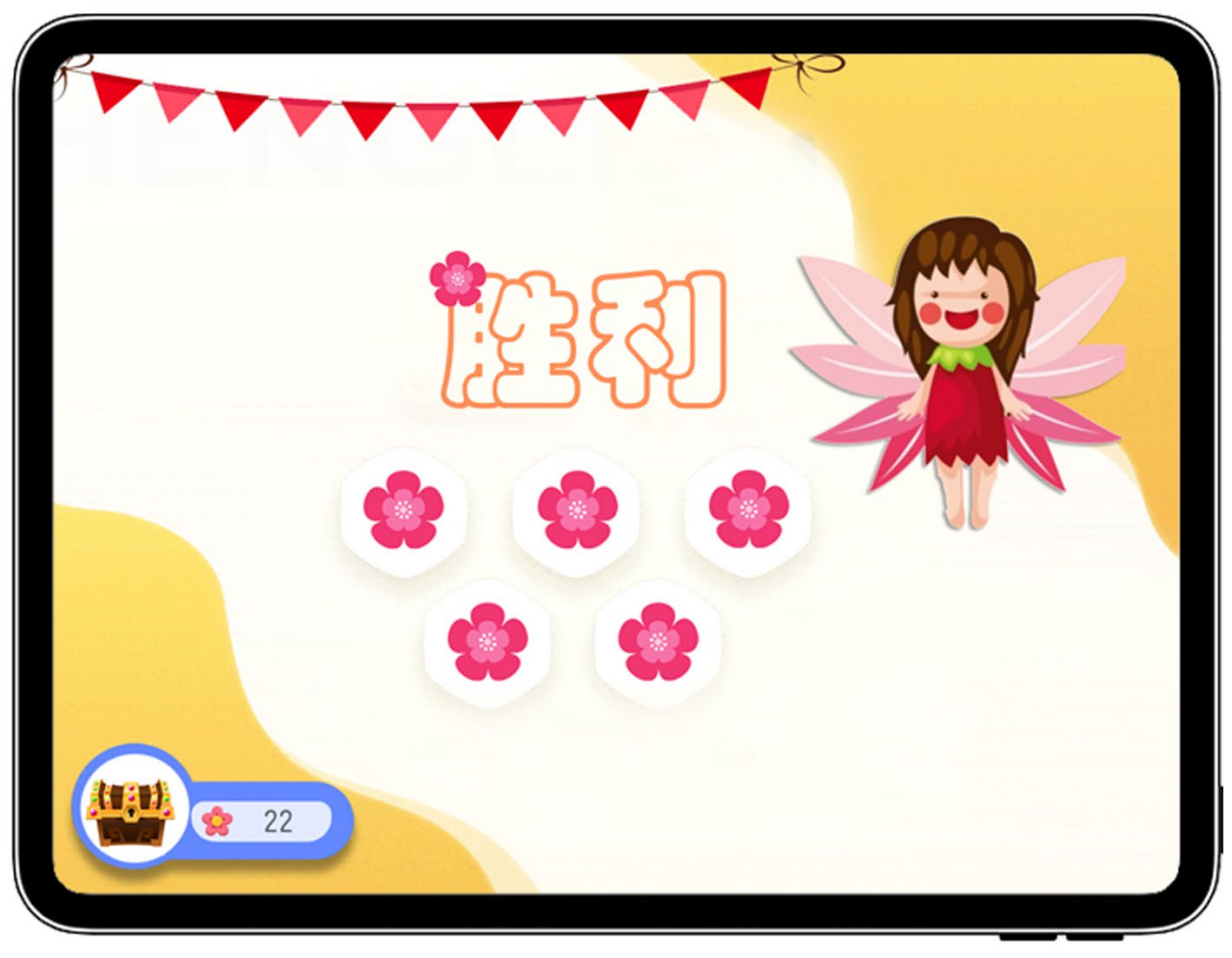
Provide feedback and rewards to students.

In addition, this GIEB provides a variety of ways for students to master Chinese characters through game play (As shown in Figure 4). For example, in the recognition stage, students can play a game to recognize the structure of Chinese characters; in the learning stage, students can deepen their knowledge of Chinese characters by moving them to the corresponding positions through a matching game; and in the writing stage, GIEB can provide students with a wireframe of Chinese characters so that they can imitate them according to the wireframe. It is important to note that in each stage, repeated practice is conducted to deepen the students’ impressions of the Chinese characters. After completing all three stages, students will be awarded a red flower as a badge to compare their Chinese character completion with other students. Students can increase their motivation to learn and feel competent by receiving rewards and positive feedback and undergoing repeated learning, which satisfies their need for competence (As shown in Figure 5).

Finally, this GIEB provides students with Chinese character completion forms and learning reports, which can be shared with their peers and encourage positive competition among students. Considering that the participants in this study were second-grade students, online features such as chat and discussion were not added because they would not be beneficial to them. It is more beneficial for them to learn using GIEB and then have class discussions, problem solving, and more practice. The need for relatedness can be satisfied by discussing problems with using the CIEB with classmates during the in-class stage, and by receiving praise from classmates and teachers for earning more badges.

Based on the three psychological needs proposed by SDT, by integrating gamification elements and story context into GIEB, we believe it has a greater motivational effect on students’ Chinese character learning.

### 3.4. Game elements in GIEB

Zainuddin et al. (2020) definition of gamified instruction as a set of instructional activities that use game-based elements and mechanics to increase student motivation and interest in learning. In addition, Buckley and Doyle (2017) identified 15 different game elements, such as achievements, avatars, badges, boss fights, gifts, and leaderboards, which are richer than the 10 game elements originally proposed by Reeves and Read (2009). For this study’s GIEB, some of these game elements were specifically selected to create a gamified learning environment that enhanced the learning content and goals. As shown in Table 2.

**Table 2:** Game elements used in the GIEB.

Game elements	Corresponding game elements	Description
Avatars	Clown Warrior	All students can control the Clown Warrior to participate in the Chinese characters Forest Challenge.
Challenge	Mini-game	Each stage has a corresponding mini-game for students to learn Chinese characters.
Feedback	Instant Feedback	Students will have instant feedback from GIEB on each learning activity.
Points and badges	Points and badges	Award small red flowers or virtual gifts; Provide learning reports

In this study, GIEB uses the story of the Clown Warrior and Fairy’s journey into the Chinese character forest to save other fairies from various challenges. To do this, each student operates their “Clown Warrior” to perform the Chinese character challenges. The games embedded in the story provide a fun learning experience for students, and the program’s various prompts and audio narration guide them to actively participate and to think independently. In addition, each Chinese character in the game’s recognition, learning, and writing phases is linked to real-life events to make learning each character fun and interesting.

For each mini-game, students can manipulate the game elements through interactive features on the GIEB interface. For example, during the learning phase, students can move the Chinese characters to the appropriate positions through the matching game; during the practice of writing, a virtual wireframe is provided for them to use, thus increasing effective interaction and learning interest. After students complete each game, GIEB provides various feedback; for example, the fairy awards a red flower badge; when students complete three Chinese characters, they receive a virtual treasure box. This can further motivate students to learn during the gamified learning process.

In summary, gamified learning content is primarily designed to increase student interest and motivation in learning. The game elements and story content promote students’ motivation and engagement in learning. By using GIEB, students are able to connect Chinese characters with things in the real world, which can deepen their comprehension and mastery of Chinese characters, and thus improve their learning performance and motivation. Therefore, this GIEB is consistent with SDT.

## 4. Materials and methods

### 4.1. Research design

The purpose of this study is to examine the effects of gamified interactive e-books on students’ learning achievements and motivation in a Chinese character learning flipped classroom. A pre-test and post-test control group experimental design was used. Each student was randomly assigned to either the experimental group or the control group by using a number generator. For this study, there were two experimental groups named GIEFC (Gamified Interactive E-book Flipped Classroom) and TFC (Traditional Flipped Classroom), while the control group used TTC (Traditional Teaching Classroom). All three groups were given pre- and post-tests, and the pre-test scores were used as covariates in the data analysis phase.

### 4.2. Participants

The participants were 90 s-grade students from a public primary school in Zhengzhou, China, who came from three different classes and shared the same Chinese teacher. The experimental school was selected by the Convenience sampling method. They had just learned Hanyu Pinyin, or Mandarin phonetic symbols, which work similarly to English phonetic symbols, in the first grade and had just started learning Chinese characters, so they had the same initial knowledge. All participants were informed prior to the experiment that they could end their participation at any time without any consequences. Moreover, informed consent forms were also given to participants by their parents or legal guardians.

The three classes were named: Class 1, Class 2, and Class 3. Based on a lottery, Class 2 was assigned as experimental group 1 (*n* = 30) and adopted the Gamified Interactive E-book Flipped Classroom (GIEFC) method and used GIEB. Class 1 was assigned as experimental group 2 (*n* = 30) and used the Traditional Flipped Classroom (TFC). Class 3 (*n* = 30) was designated as the control group and used the Traditional Teaching Classroom (TTC).

### 4.3. Instruments

The measurement instruments used in this study included the Chinese Characters Pre-test, Chinese Characters Post-test, and Electronic Storybook Motivation Scale (ESMS).

Chinese characters were tested using the method used in Wang and Yang’s (2016) study. The Chinese character pre-test and post-test consisted of 16 items each. The test included six fill-in-the-blank questions (6*2 = 12 points) to assess Chinese character writing, five single-choice questions to assess lexical comprehension (5*2 = 10 points), and five matching questions to assess lexical usage (5*2 = 10 points). The total score of the test is 32 points. To answer the fill-in-the-blank questions, students had to read a Chinese phrase containing a target Chinese character along with a Mandarin phonetic symbol and then write the corresponding Chinese character. For the single-choice and matching questions, students were provided with pictures of the target Chinese character. For example, if the target word was sun, students were provided with a picture of the sun. Students had to identify the pronunciation and structure of a word before selecting the most appropriate answer. All questions are designed based on the six target characters. The pre- and post-tests are similar in format and difficulty. Each student is given the scores of the three items after the test, and the scores are added together to give them a final score for their learning achievement. The test items were validated by three experienced Chinese language teachers for expert validity (Yang and Chan, 2008).

The instrument used in this study to assess student motivation was adapted from the Electronic Storybook Motivation Scale (ESMS), which was previously used by Kao et al. (2016) in their research. The original ESMS contains thirty questions, and its Cronbach’s alpha is 0.88, while the Cronbach alpha for each sub-dimension is 0.82–0.93. This indicates the high reliability of the scale. Two of the dimensions, intrinsic and extrinsic motivation, were selected for this study, which is consistent with the SDT classification of motivation. The intrinsic motivation section included five questions, for example, question 1:“I think learning Chinese characters is very interesting and will improve my knowledge.” The extrinsic motivation section also included five questions, for example, question 7: “Whether I like learning Chinese characters or not, I do not want to disappoint my teacher by not learning well.” A Likert-type scale ranging from 1 (strongly disagree) to 5 (strongly agree) was used for this questionnaire. The scores range from a minimum of 10 (1*10 = 10) to a maximum of 50 (5*10 = 50). The higher the score, the more motivation the student has to learn. The Cronbach’s alpha value of the ESMS used in this study is 0.85.

### 4.4. Experimental procedure

**Figure 6:**
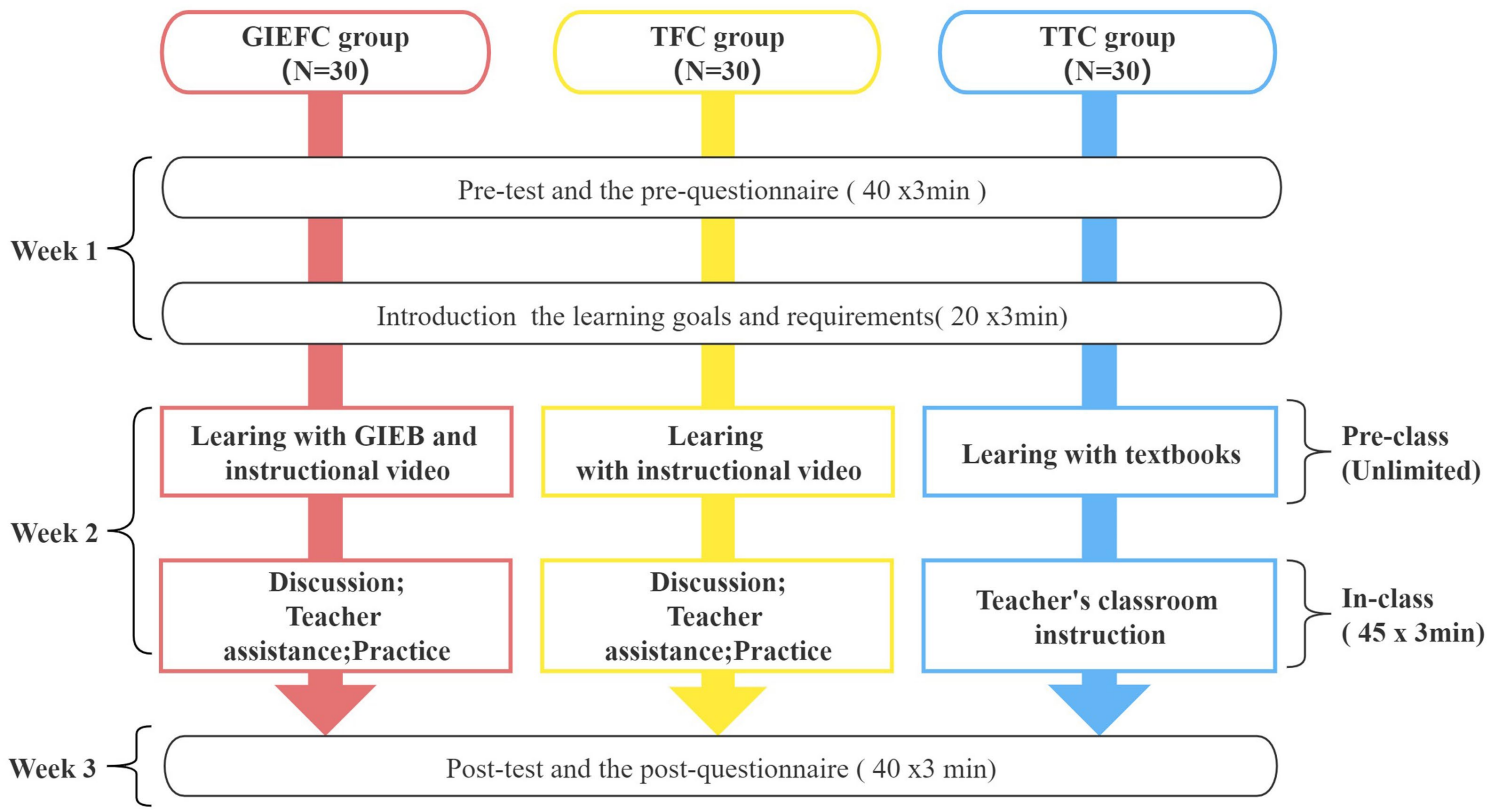
Experimental procedure.

The duration of this experiment is three week. Before the experiment, all students were required to take the Chinese character pre-test and the pre-motivation questionnaire (ESMS) for a total of 40 min. Then, the teacher gave a 20-min introduction the learning goals and requirements. In the second week, students carry out learning activities based on their group’s learning strategies. Post-test and post-motivation questionnaire were (ESMS) conducted in the third week. Figure 6 illustrates the experimental procedure.

The GIEFC group uses GIEB as a learning tool to learn Chinese characters in the pre-class stage, and a 20-min instructional video is also provided to them. During the in-class stage, they discuss the problems they have encountered in the pre-class stage with their classmates, receive assistance from the teacher, and engage in hands-on activities. The TFC group has the same activities as the GIEFC group except that they do not use the GIEB. The control group, or TTC group, uses their textbooks for the pre-class stage study, and during the in-class stage, teachers mainly teach through traditional teaching methods. Students in both experimental and control groups spent 45 min in class, as the standard class time in Chinese primary schools is 45 min, with four classes per half day. It is necessary to emphasize that students could study repeatedly as needed during the pre-class stage. After the learning activities, there was a Chinese character post-test and a post-motivation questionnaire (ESMS) for a total of 40 min.

## 5. Data analysis and results

In this study, analysis of covariance (ANCOVA) was used to test the differences between the pre-test and post-test scores of the experimental and control groups. The pre-test scores of both the experimental and control groups were also used as covariates, which eliminated the effect of the pre-test scores on the post-test scores and made the data from the experiment more sensitive and accurate. To use ANCOVA, the following assumptions must be fulfilled: (1) Normality: the dependent variable must be continuous and normally distributed within each group; (2) Homogeneity of variance: the variance of the dependent variable should be equal across groups; (3) Homogeneity of regression slope: the slope of the regression line should be equal across groups. All data were calculated using SPSS 26 (Statistical Package for the Social Sciences 26) and tested at a significance level of 0.05.

### 5.1. Learning achievement

This section focuses on the analysis of students’ achievements in Chinese character learning, using students’ pre-test scores as a covariate. The Kolmogorov–Smirnov test was used to test the normality distribution of the data, and the results showed (Kolmogorov–Smirnov = 0.22, *p* > 0.05) that the post-test scores were within the normality distribution. In addition, the results of Levene’s test of homogeneity of variance (*p* > 0.05) and the results of homogeneity of regression slopes (*F* = 2.27, *p* = 0.15 > 0.05) are also fulfilled. Therefore, the ANCOVA was performed.

Table 3 shows the results of descriptive data and ANCOVA for the Chinese character test based on the students’ post-test scores in the three groups. The adjusted means for the three groups were 21.36 for the GIEFC group, 18.54 for the TFC group, and 18.16 for the TTC group. From the ANCOVA results, there was a statistically significant difference between the post-test scores of the three groups (*F* = 16.97, *p* < 0.001). To determine the specific differences between each group, a *post hoc* analysis was also conducted, and an LSD test revealed that the GIEFC group scored significantly higher (21.36) than the TFC group (18.54) and the TTC group (18.16) (*p* < 0.05), while there was no statistical difference between the scores of the TFC and TTC groups (*p* > 0.05). This result indicates that the use of GIEB-based Chinese character learning flipped classroom had better learning achievement than those using traditional flipped classroom and traditional teaching classroom, while there was no difference between the traditional flipped classroom method and the traditional teaching method.

**Table 3:** Descriptive data and ANCOVA result of the learning achievement post-test by group.

Variable	Group	*N*	Mean	S.D	Adjusted Mean	*F*	*Post Hoc*
Post-test	(1) GIEFC	30	21.20	2.66	21.36	16.97***	(1) > (2)
	(2) TFC	30	18.53	2.91	18.54		(1) > (3)
	(3) TTC	30	18.33	2.78	18.16		

***p < 0.001.

To further analyze the differences in Chinese character writing, lexical comprehension, and lexical usage among the three groups, ANCOVA was performed on these three items. The results of the Kolmogorov–Smirnov test were Chinese character writing (Kolmogorov–Smirnov = 0.25, *p* > 0.05), lexical comprehension (Kolmogorov–Smirnov = 0.53, *p* > 0.05), and lexical usage (Kolmogorov–Smirnov = 0.27, *p* > 0.05). The results of Levene’s test for homogeneity of variance all fulfilled the ANCOVA hypothesis (*p* > 0.05). In addition, the results of homogeneity of regression slopes were also fulfilled with Chinese character writing (*F* = 0.25, *p* > 0.05), lexical comprehension (*F* = 4.2, *p* > 0.05), and lexical usage (*F* = 1.03, *p* > 0.05). The above results indicate that all three items can be analyzed using ANCOVA.

From the results in Table 4, it was found that all three groups showed significant differences in the ANCOVA results on the three items, with Chinese character writing (*F* = 18.89, *p* < 0.001), lexical comprehension (*F* = 4.06, *p* < 0.05), and lexical usage (*F* = 6.84, *p* < 0.05). The LSD results showed that the students in the GIEFC group had a significantly higher adjusted mean (7.58) than the TFC group (6.22) and the TTC group (6.14) for the Chinese character writing, while there was no difference between the TFC (6.22) and TTC (6.14) groups. For the lexical comprehension item, the GIEFC group still scored significantly higher adjusted mean (6.74) than the TFC group (6.06) and the TTC group (6.13), although the adjusted mean of the TTC group (6.13) was slightly higher than the TFC group (6.06), but this was not a significant difference (*p* > 0.05). Finally, there were different results in the lexical usage item, with students in the GIEFC group having a significantly higher adjusted mean (7.28) than the TFC group (6.21) and the TTC group (6.62), while the adjusted mean of the TTC group (6.62) and the TFC group (6.21) formed a significant statistical difference (*p* < 0.05). In other words, the students in TTC group (6.62) performed better than TFC group (6.21) in terms of lexical usage. In conclusion, the use of the GIEB-based Chinese character learning flipped classroom (GIEFC group) showed superior results compared to the other two learning approaches.

**Table 4:** Descriptive data and ANCOVA results of the post-test scores for the three items by group.

Items	Group	*N*	Mean	S.D	Adjusted Mean	*F*	*Post Hoc*
Chinese character writing	(1) GIEFC	30	7.53	1.54	7.58	18.89***	(1) > (2)
	(2) TFC	30	6.27	1.55	6.22		(1) > (3)
	(3) TTC	30	6.13	1.38	6.14		
lexical comprehension	(1) GIEFC	30	6.70	0.76	6.74	4.06*	(1) > (2)
	(2) TFC	30	6.07	0.36	6.06		(1) > (3)
	(3) TTC	30	6.18	0.36	6.13		
lexical usage	(1) GIEFC	30	7.27	1.23	7.28	6.84*	(1) > (2)
	(2) TFC	30	6.20	1.42	6.21		(1) > (3)
	(3) TTC	30	6.63	1.38	6.62		(3) > (2)

*p < 0.05; ***p < 0.001.

### 5.2. Learning motivation

This section analyzes students’ motivation to learn Chinese characters, using their scores on the pre-test of the post-motivation questionnaire (ESMS) as a covariate. Before running ANCOVA, to ensure that the assumptions for using ANCOVA were fulfilled, the post-test scores on learning motivation were tested. The normality distribution was tested on the post-test scores of learning motivation, and the results showed that the scores were within a normal distribution (Kolmogorov–Smirnov = 0.16, *p* > 0.05). The results of Levene’s test of homogeneity of variance (*p* > 0.05) and the results of homogeneity of regression slopes (*F* = 0.67, *p* = 0.52 > 0.05) are also satisfied, which means that ANCOVA can be performed.

The results of the ANCOVA are summarized in Table 5. A significant difference in the post-test scores of motivation can be found among the three groups of students (*F* = 63.34, *p* < 0.001). *Post hoc* analysis revealed that the students in the GIEFC group had an adjusted mean of 39.14, significantly higher than the TFC group (34.80) and the TTC group (34.27). This result suggests that the use of GIEB-based Chinese language flipped classroom approach (GIEFC group) had better learning motivation than those using traditional flipped classroom approach and traditional teaching classroom approach.

**Table 5:** Descriptive data and ANCOVA result of the learning motivation post-test by group.

Variable	Group	*N*	Mean	S.D	Adjusted Mean	*F*	*Post Hoc*
Post-test	(1) GIEFC	30	38.90	3.01	39.14	63.34***	(1) > (2)
	(2) TFC	30	35.03	2.13	34.80		(1) > (3)
	(3) TTC	30	34.27	1.70	34.27		

***p < 0.001.

To further investigate the differences between students’ intrinsic motivation and extrinsic motivation during Chinese character learning, ANCOVA was used to analyze these two types of motivation. Students’ pre-tests of intrinsic motivation and extrinsic motivation were used as covariates. In testing whether the data fulfilled the assumptions of using ANCOVA, it was found that the results of the normality distribution test were: intrinsic motivation (Kolmogorov–Smirnov = 0.18, *p* > 0.05) and extrinsic motivation (Kolmogorov–Smirnov = 0.21, *p* > 0.05). The results of Levene’s test for homogeneity of variance all satisfied the ANCOVA hypothesis (*p* > 0.05), and the homogeneity of regression slopes was confirmed: intrinsic motivation (*F* = 1.15, *p* > 0.05) and extrinsic motivation (*F* = 0.22, *p* > 0.05). The above test results indicate that ANCOVA can be used.

As shown in Table 6, there were significant differences among the three groups in terms of students’ intrinsic motivation (*F* = 12.79, *p* < 0.001) and extrinsic motivation (*F* = 39.27, *p* < 0.001) for the learning process. Specifically, the adjusted mean for intrinsic motivation showed that the GIEFC group (19.07) scored significantly higher than the TFC group (17.60) and the TTC group (16.82), while there was no significant difference between the TFC and TTC groups. For the adjusted mean of extrinsic motivation, students in the GIEFC group (19.97) still performed better than the other two groups; students in the TFC group had higher extrinsic motivation scores (18.23) than those in the TTC group (17.44). This indicates that students using the GIEB-based Chinese language flipped classroom approach (GIEFC group) performed better than the other two groups in terms of intrinsic and extrinsic motivation to learn Chinese characters.

**Table 6:** Descriptive data and ANCOVA results of the post-test scores for the two motivation by group.

Motivation Type	Group	*N*	Mean	S.D	Adjusted Mean	*F*	*Post Hoc*
Intrinsic motivation	(1) GIEFC	30	18.97	2.33	19.07	12.79***	(1) > (2)
	(2) TFC	30	17.63	1.38	17.60		(1) > (3)
	(3) TTC	30	16.83	1.30	16.82		
Extrinsic motivation	(1) GIEFC	30	19.93	1.44	19.97	39.27***	(1) > (2)
	(2) TFC	30	18.20	1.38	18.23		(1) > (3)
	(3) TTC	30	17.43	1.04	17.44		(2) > (3)

***p < 0.001.

## 6. Discussion and conclusion

### 6.1. Discussion

This study proposed the Gamified Interactive E-book-Based Flipped Classroom (GIEFC) method and developed a Gamified Interactive E-book (GIEB) to enhance students’ motivation and learning achievements in Chinese character learning. 90 students from a second-grade primary school in Zhengzhou, China, were randomly assigned to one of two experimental groups (the GIEFC and TFC groups) and one control group (the TTC group).

In terms of students’ Chinese character learning performance, students in the GIEFC group performed significantly better than those in the TFC and TTC groups. This result is consistent with the findings of Zainuddin’s (2018) study that the gamified flipped classroom approach promotes better motivation and engagement, especially in the gamified learning process where students are rewarded for their learning and compete with other students. It should be noted that there was no difference between the Chinese character learning performance of students in the TFC group and those in the TTC group, which is consistent with the findings of Yang et al. (2018), Yong et al. (2015), and Abeysekera and Dawson (2015), who found no differences in students’ academic performance between the flipped approach and the traditional models. In the traditional flipped classroom, the quality of the pre-course learning materials provided by the teacher and the availability of effective learning strategies for students’ pre-class learning may affect their motivation and learning outcomes.

This study also analyzed three items of Chinese character learning and found that students in the GIEFC group performed significantly better than the TFC and TTC groups in Chinese character writing, lexical comprehension, and lexical usage. This result suggests that gamified interactive e-books designed based on SDT provide students with game elements that match the learning content, and students are more motivated to participate in the gamified learning process, thus improving their learning performance (Huang et al., 2022). Buckley and Doyle (2016) focused on learning styles and personalities along with gamified teaching strategies. The results indicated that students’ engagement, attitudes, and performance had developed and improved. Gamified interactive e-books provide more interactive and practical opportunities for students to learn through games and story contexts, thus expanding their knowledge of Chinese characters. For example, in the Chinese character writing part, the gamified interactive e-book provides students with a wireframe of Chinese characters that they can practice writing by following the instructions on a touch screen. At the same time, the story context connects real-world objects to characters, which can enhance students’ ability to understand and use words.

In terms of learning motivation, students in the GIEFC group scored significantly higher than the other two groups in terms of both intrinsic and extrinsic motivation. This finding is consistent with Barata et al.'s (2017) findings that gamification can make teaching and learning more engaging and encourage more active work through badges, scores, and leaderboards. The gamified interactive e-books developed and used in this study were developed based on SDT, which emphasizes people’s three main psychological needs (i.e., competence, autonomy, and relatedness). Deci and Ryan’s (2008) study showed that meeting people’s psychological needs can sustain intrinsic motivation and regulate extrinsic motivation. Adukaite et al.’s (2017) and Jurgelaitis et al.’s (2019) studies also claim that gamification has a positive effect on both extrinsic and intrinsic motivation. Furthermore, this positive result is mainly due to the gamified interactive e-book developed in this study, which implements gamification that creates a positive, intrinsically motivating, and “gameful” experience and incorporates extrinsic rewards and intrinsic satisfaction (Ryan and Deci, 2002). These game elements include avatars, challenges, feedback, points, and badges that serve to present Chinese character learning in a gamified and story-driven context. As a result, student motivation and engagement are increased, which ultimately leads to the achievement of learning goals (Lo and Hew, 2020). It is important to note that the effective design and implementation of gamification strategies must take into account the learning objectives and align them with students’ intrinsic motivation (da Rocha Seixas et al., 2016).

It is worth noting that students in the TFC group scored higher than those in the TTC group in terms of their extrinsic motivation, indicating that they are interested in new teaching methods that promote their extrinsic motivation. However, it is surprising that there is no difference between the TFC and TTC groups in terms of intrinsic motivation. The possible reasons for this could be that the pre-class learning materials provided by the traditional flipped classroom are unattractive and do not stimulate students’ interest but instead become an extra workload, or that students have difficulty understanding the learning content at home before class. This may lower students’ intrinsic motivation and lead them to not learn enough during the in-class phase, which ultimately leads to poor learning outcomes (Jensen et al., 2018; Tse et al., 2019). This is consistent with the study by Awidi and Paynter (2019), who found that the flipped classroom method was effective in creating significant extrinsic motivation in students but had no effect on intrinsic motivation (Awidi and Paynter, 2019).

### 6.2. Conclusion

This study focuses on the effects of gamified interactive e-books on students’ learning achievements and motivation in a Chinese character learning flipped classroom. The results show that the use of gamified interactive e-books in the flipped classroom has a positive effects on students’ motivation and achievements in learning Chinese characters.

From a practical perspective, the results of this study demonstrate the feasibility of using self-determination theory (SDT) to design gamified interactive e-books, which can provide an idea for teachers and designers. In addition, this study provides another proof of the effectiveness of using educational technology in cooperation with the flipped classroom. Finally, the results of this study can encourage teachers to actively investigate the extension of gamified interactive e-books to flipped classroom practices in other subjects to help students overcome the challenges they encounter in learning. For example, a meta-analysis conducted by Wijaya et al. (2022a,b) found that the use of mathematics e-books had a significant effect on students’ mathematics achievement.

Theoretically, the effectiveness of the gamified interactive e-book used in this study confirms the validity of self-determination theory (SDT). In addition, this study provide additional evidence for the validity of the flipped classroom pedagogy. Finally, this study also demonstrates the effectiveness of using self-designed gamified interactive e-books in the flipped classroom.

## 7. Limitation and recommendation

Like other empirical studies, this study has its limitations. First, the participants in this study were 90 s-grade students from a public primary school in Zhengzhou, China, so the results of this study cannot be generalized to a national context. Second, the sample size of this study was not large enough, and future studies could use a larger number of participants, even students from different schools; the duration of this study was short-term, and future studies could conduct long-term experiments. Finally, in the process of designing the gamified interactive e-book, only some game elements were selected, and future studies can explore adding more game elements, such as leaderboards, quizzes, and other elements. It should be reminded that the design of gamified interactive e-books may increase the workload of teachers, so researchers should provide the necessary support to ensure that they can master the design process. Future research needs to incorporate the factors that influence teachers’ use of gamified interactive ebooks into the research aims (Wijaya et al., 2022a,b).

## Data availability statement

The raw data supporting the conclusions of this article will be made available by the authors, without undue reservation.

## Ethics statement

The studies involving human participants were reviewed and approved by Ethics Committee of School of Art and Design, Zhengzhou University of Industrial Technology. The patients/participants provided their written informed consent to participate in this study. Written informed consent was obtained from the individual(s) for the publication of any identifiable images or data included in this article.

## Author contributions

CC, NJ, and YM contributed to the design and implementation of the research, the analysis of the results, and writing of the manuscript. All authors contributed to the article and approved the submitted version.

## Conflict of interest

The authors declare that the research was conducted in the absence of any commercial or financial relationships that could be construed as a potential conflict of interest.

## Publisher’s note

All claims expressed in this article are solely those of the authors and do not necessarily represent those of their affiliated organizations, or those of the publisher, the editors and the reviewers. Any product that may be evaluated in this article, or claim that may be made by its manufacturer, is not guaranteed or endorsed by the publisher.
