# Supplementary material for: The study on the effects of gamified interactive e-books on students’ learning achievements and motivation in a Chinese character learning flipped classroom
Source: Front Psychol. 2023 Aug 4;14:1236297. doi: 10.3389/fpsyg.2023.1236297 (PMC10438992; doi:10.3389/fpsyg.2023.1236297)
Supplement: Supplementary file 1 [file Data_Sheet_1.PDF]

The Chinese character pre-test

Part1: fill-in-the-blank questions(6*2 = 12 points)

dì yī bù fēn： tián kōng tí
第一部分：填空题

1. There are all kinds of______on the road.(cars)

mǎ lù shàng yǒu gè zhǒng gè yàng de chē （chē）
马路上有各种各样的____。（车）

2. After school, many children are playing in front of the_______ .(door)

fàng xué hòu， hěn duō dōu xiǎo péng yǒu dōu zài mén qián wán。（mén）
放学后，很多都小朋友都在_____前玩。（门）

3. There are a lot of _______ on the farm.(cows)

nóng chǎng lǐ yǒu hěn duō de niú。（niú）
农 场里有很多的_____。（牛）

4. The______ couldn't find its mother.(sheep)

nà zhī yáng zhǎo bú dào tā de mā mā le。（yáng）
那只_____找不到它的妈妈了。（羊）

5. It was raining and the children went home with________.(umbrellas)

xià yǔ le， xiǎo péng yǒu men dōu dǎ zhe sǎn huí jiā。（sǎn）
下雨了，小朋友们都打着_____回家。（伞）

6. We need to develop a good habit of brushing our________every day.(teeth)

wǒ mén yào yǎng chéng měi tiān shuā yá de hǎo xí guàn。（yá）
我们要养成每天刷_____的好习惯。（牙）

Part 2: single-choice questions(5 *2 = 10 points)

dì bù fēn： dān xiàng xuǎn zé tí
第2部分：单项选择题

1. Which of the following is a car?

xià miàn nǎ yí gè shì qì chē？
下面哪一个是汽_____？

A 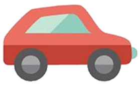 B 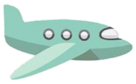 C 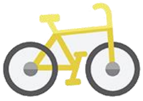

2. Which of the following animals is a cow?

xià miàn nǎ yí gè dòng wù shì niú？
下面哪一个动物是_____？

A 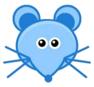 B 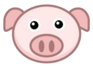 C 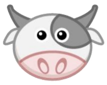

3. Which of the following animals is a sheep?

xià miàn nǎ yí gè dòng wù shì yáng？
下面哪一个动物是_____？

A 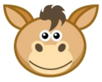 B 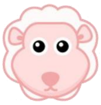 C 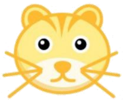

4. Which of the following items is a umbrella？

xià miàn nǎ yí gè wù pǐn shì sǎn
下面哪一个物品是_____？

A 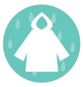 B 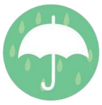 C 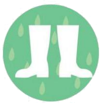

5. Which of the following pictures are teeth？

xià miàn nǎ yí gè tú piàn shì yá
下面哪一个图片是_____？

A 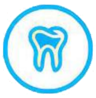 B 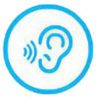 C 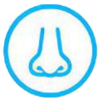

Part 3: Matching (5 *2 = 10 points)

dì bù fēn lián xiàn tí
第3部分：连线题

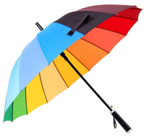

mén

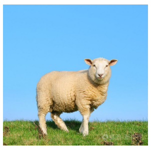

chē

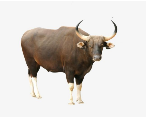

niú

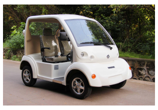

sǎn

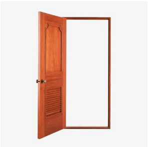

mǎ

The Chinese character post-test

Part1: fill-in-the-blank questions(6*2 = 12 points)

dì yī bù fēn tián kōng tí
第一部分：填空题

1. We have to be a good______ .(person)

wǒ mén yào zuò yí gè shàn liáng de rén rén
我们要做一个善良的_____。（人）

2. We can't put toys in our _______。（mouth）

wǒ mén bù néng jiāng wán jù fàng jìn kǒu zhōng kǒu
我们不能将玩具放进_____中。（口）

3. Each child has two_______(hands)

měi ge xiǎo péng yǒu dōu yǒu liǎng zhī shǒu shǒu
每个小朋友都有两只_____。（手）

4. The_____is red, big and round in the mornin.(sun)

zǎo shàng de rì shì hóng sè de yòu dà yòu yuán rì
早上的_____是红色的，又大又圆。（日）

5. The_____at the Mid-Autumn Festival is the largest, roundest and brightest.(moon)

zhōng qiū jié de yuè yuè
中秋节的_____亮是最大最圆和最亮的。(月)

6. There were many dark_____in the sky and it was going to rain soon.(clouds)

tiān kōng zhōng chū xiàn le hěn duō wū yún yún
天空中出现了很多乌_____，马上就要下雨了。(云)

Part 2: single-choice questions(5 *2 = 10 points)

dì bù fēn dān xiàng xuǎn zé tí
第2部分：单项选择题

1. Which of the following is sun?

xià miàn nǎ yí gè shì rì
下面哪一个是_____？

A 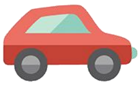 B 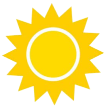 C 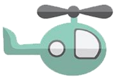

2. Which of the following pictures represents hand?

xià miàn nǎ yí gè tú piàn shì ~~shǒu~~
下面哪一个图片是——？

A 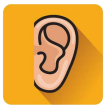 B 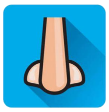 C 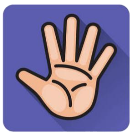

3. Which of the following pictures represents cloud?

xià miàn nǎ yí gè tú piàn shì yún
下面哪一个图片是_____？

A 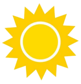 B 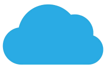 C 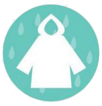

4. Which of the following pictures represents a people?

xià miàn nǎ yì zhāng tú piàn shì rén
下面哪一张图片是_____？

A 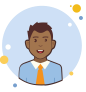 B 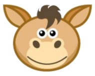 C 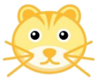

5. Which of the following pictures represents the mouth?

xià miàn nǎ yí gè tú piàn shì kǒu
下面哪一个图片是_____？

A 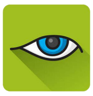 B 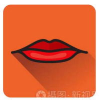 C 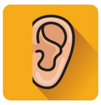

Part 3: Matching (5 *2 = 10 points)

dì bù fēn lián xiàn tí
第3部分：连线题

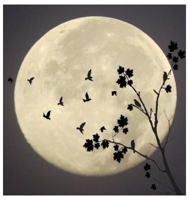 kǒu

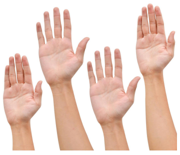 rì

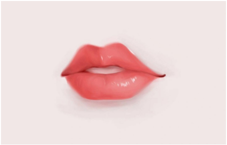 yuè

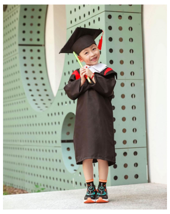 shǒu

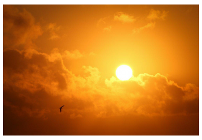 rén

Electronic Storybook Motivation Scale

(The instrument used in this study to assess student motivation was adapted from the Electronic Storybook Motivation Scale (ESMS), which was previously used by Kao, Tsai, Liu, and Yang (2016) in their research.)

1 = Strongly Disagree;
2 = Disagree;
3 = Neither Agree nor Disagree;
4 = Agree;
5 = Strongly Agree

NO.	ITEMS	POINTS				
1	I think learning Chinese characters is very interesting and will improve my knowledge.	1	2	3	4	5
2	Learning Chinese characters can make me a useful person.	1	2	3	4	5
3	When I took the course, sometimes I felt very happy and satisfied.	1	2	3	4	5
4	When I was studying this course, I felt that my knowledge was increasing and I felt relaxed and happy.	1	2	3	4	5
5	After completing this course, I will continue to learn more about it, which makes me feel satisfied and I have a sense of pride after each study.	1	2	3	4	5
6	My parents would be proud of me if I could get a good grade in this class	1	2	3	4	5
7	Whether I like learning Chinese characters or not, I don't want to disappoint my teacher by not learning well.	1	2	3	4	5
8	If I know I get bad grades in the course, I will be afraid about my performance in the next test.	1	2	3	4	5
9	I know having good performance in the course can help me get a good job in the future.	1	2	3	4	5
10	If I can get good grades in this class, other students will envy me.	1	2	3	4	5
Sample No.____________		Total points__________				
